# Modulation of the Gut Microbiota in Memory Impairment and Alzheimer’s Disease via the Inhibition of the Parasympathetic Nervous System

**DOI:** 10.3390/ijms232113574

**Published:** 2022-11-05

**Authors:** Sunmin Park, Xuangao Wu

**Affiliations:** 1 Department of Bioconvergence, Hoseo University, Asan 31499, Korea; 2 Department of Food and Nutrition, Obesity/Diabetes Research Center, Hoseo University, Asan 31499, Korea

**Keywords:** gut dysbiosis, parasympathetic nervous system, mild memory impairment, Alzheimer’s disease, scopolamine

## Abstract

The gut microbiota has been demonstrated to play a critical role in maintaining cognitive function via the gut-brain axis, which may be related to the parasympathetic nervous system (PNS). However, the exact mechanism remains to be determined. We investigated that patients with mild cognitive impairment (MCI) and Alzheimer’s disease (AD) could exhibit an altered gut microbiota through the suppression of the PNS, compared to the healthy individuals, using the combined gut microbiota data from previous human studies. The hypothesis was validated in rats to suppress the PNS by scopolamine injections. The human fecal bacterial FASTA/Q files were selected and combined from four different AD studies (n = 410). All rats had a high-fat diet and treatments for six weeks. The MD rats had memory impairment by scopolamine injection (2 mg/kg body weight; MD, Control) or no memory impairment by saline injection. The scopolamine-injected rats had a donepezil intake as the positive group. In the optimal model generated from the XGboost analysis, *Blautia luti, Pseudomonas mucidoiens, Escherichia marmotae,* and *Gemmiger formicillis* showed a positive correlation with MCI while *Escherichia fergusonii*, *Mycobacterium neglectum,* and *Lawsonibacter asaccharolyticus* were positively correlated with AD in the participants with enterotype Bacteroides (ET-B, n = 369). The predominant bacteria in the AD group were negatively associated in the networking analysis with the bacteria in the healthy group of ET-B participants. From the animal study, the relative abundance of *Bacteroides* and *Bilophilia* was lower, and that of *Escherichia*, *Blautia*, and *Clostridium* was higher in the scopolamine-induced memory deficit (MD) group than in the normal group. These results suggest that MCI was associated with the PNS suppression and could progress to AD by exacerbating the gut dysbiosis. MCI increased *Clostridium* and *Blautia,* and its progression to AD elevated *Escherichia* and *Pseudomonas*. Therefore, the modulation of the PNS might be linked to an altered gut microbiota and brain function, potentially through the gut-brain axis.

## 1. Introduction

With an increase in the life expectancy, cognitive impairment is a significant health problem, worldwide. Mild cognitive impairment (MCI) is an early stage of loss of memory or cognitive function in people unable to perform independent daily activities [1]. The causes for the development of MCI have not been completely elucidated; however, it is known that it is related to changes in the brain during the early stages of neurodegenerative diseases, including Alzheimer’s disease (AD) [1]. People with MCI are more susceptible to dementia, including AD. About 10–20% of people, aged over 65 years with MCI develop dementia over one year, and about 80% progress to AD, as seen in a 6-year follow-up study [2]. Therefore, MCI can be considered a pre-dementia stage that can progress to AD.

AD is the most common form of dementia in people in their 70s and 80s, and its prevalence doubles every five years after 90 years old [3]. The incidence of AD is higher in women than men, which may be attributed to their increased longevity. The pathophysiology of AD still needs to be completely understood. Current data suggest that neuronal cell death with the accumulation of amyloid-β (1–42) results in neurodegenerative changes in the brain [4]. Insoluble amyloid-β (1–42) is derived from amyloid precursor protein (APP) cleaved by β- and γ-secretases and deposited in the brain. The amyloid-β deposition by neurofibrillary tangles is involved in the tau phosphorylation [4]. In addition, the AD etiology is involved in proinflammatory processes to induce brain damage [5] and neuronal cell biosynthesis by changing the expression of various neurotrophic factors, such as the brain-derived neurotrophic factors (BDNFs) and ciliary neurotrophic factors (CNTFs), and their receptor activities [6]. Memory dysfunction is also associated with the loss of cholinergic neurons linked to an acetylcholine deficiency [7]. The current opinion is that the acetylcholine dysfunction is not a pathological cause for AD but its consequence [8]. Acetylcholinesterase inhibitors, such as donepezil, elevate the acetylcholine availability at the synapses in the brain to improve cognitive function [9]. Furthermore, the vagus nerve stimulation is a potential therapy for MCI and early-stage AD [10].

Acetylcholine is the primary neurotransmitter of the parasympathetic nervous system (PNS). The vagus nerve is the principal component of the PNS [9] and the primary connection between the brain and the gastrointestinal tract (GIT). It sends information about the status of the organs in the digestive tract to the brain via afferent fibers [10]. Treatments that target the vagus nerve system increase the vagal tone and inhibit the cytokine production [9]. Vagus nerve activation inhibits the peripheral inflammation and decreases the intestinal permeability. By contrast, stress suppresses the vagus nerve, modulates the gut microbiota, and is involved in the pathophysiology of gastrointestinal disorders through inflammatory processes [11]. Therefore, the vagus nerve acts as the bidirectional modulator of the brain-gut axis in patients with AD and other inflammatory conditions and may be associated with modulating the composition of the gut microbiota to dysbiosis or eubiosis [11].

It is difficult to determine which gut microbes play a critical role in the gut-brain axis. Based on the bacterial composition of the gut microbiota, healthy humans are categorized into three distinct enterotypes by a principal component analysis (PCA): Bacteroides, Ruminococcus, and Prevotella [12]. The gut microbiota composition of each enterotype is different and includes bacteria that can co-exist. The dietary intake and lifestyles of individuals also influence the microbes leading to different enterotypes. However, the disturbance of the vagus nerve system brings major changes in the gut microbiota via the bidirectional modulation of the gut-microbiota-gut-brain axis [13,14], and the changes may be different, according to the different enterotypes. Memory impairment is also linked to a disturbance of the vagus nerve system [9]. Therefore, AD and MCI may influence the modulation of the gut microbiota composition by altering the host’s vagus nerve system. The present study hypothesized that MCI and AD patients exhibit an altered gut microbiota, compared to healthy individuals, possibly through the PNS in those with the Bacteroides enterotype. The hypothesis was examined, using combined gut microbiota data from previous human studies and validated in rats with memory impairment induced by scopolamine injections to suppress the PNS. This study could show that gut bacteria are related to MCI and AD and outline their functions affecting the gut microbiota-gut-brain axis. It could suggest potential therapies for modulating the axis.

## 2. Results

### 2.1. Included Human Studies and Participants’ Enterotypes, According to the PCA

Four studies (PRJEB32675, PRJNA489760, PRJNA496408, and PRJNA734525) related to memory deficit were selected, and they included healthy, MCI, and AD categories. PRJNA489760 and PRJNA496408 were conducted on Chinese individuals aged 63–77 years. PRJEB32675 and PRJNA496408 were performed in Singapore and Turkey on participants aged 60–85 (Table 1). The gut microbiota is modified with not only genetic differences, such as ethnicity, but also environmental factors, such as diet, exercise, energy status, alcohol consumption, smoking, and disease status [15,16]. Although the samples mainly originated from Asians, the enterotypes were different in healthy participants, due to the different environmental factors. Supplemental Figure S1. However, most participants belonged to the enterotype Bacteroides (ET-B).

**Figure 1:**
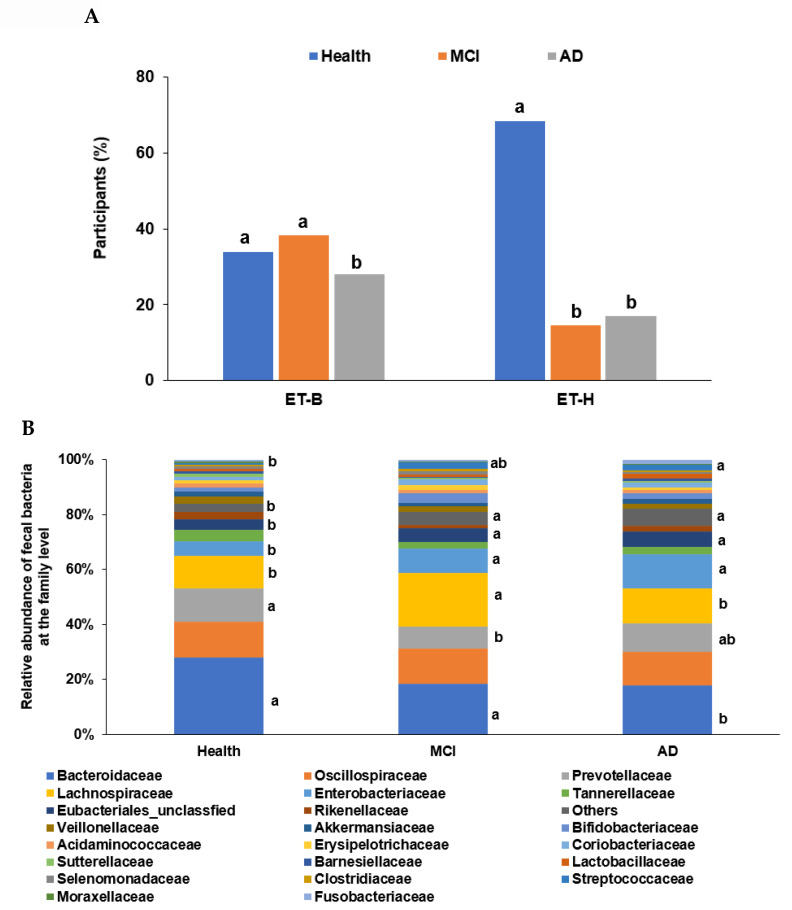

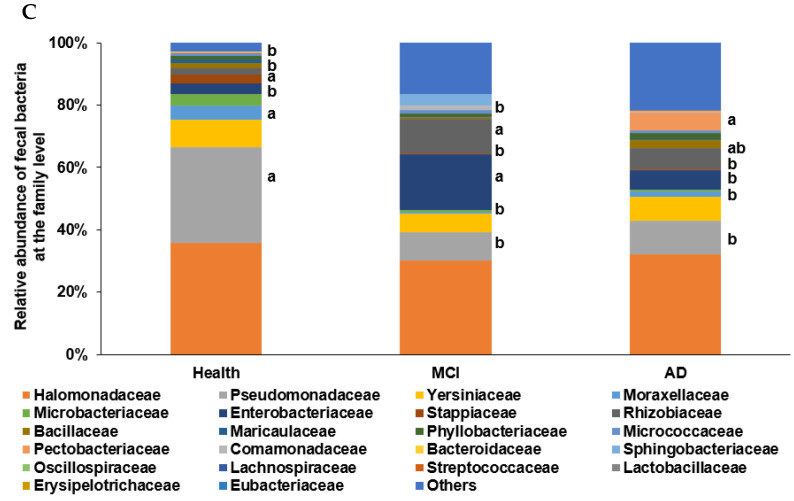
Characteristics of the gut microbiota from the human fecal bacteria of all participants. (**A**). Relative abundance of the fecal bacteria at the family level in the enterotype Bacteroides. (**B**). Relative abundance of the fecal bacteria at the family level in the enterotype Halomonas (ET-H). (**C**) The participants, according to enterotype Bacteroides (ET-B) and ET-H. ^a,b^ Different letters on the bar indicated significant differences among the groups at *p* < 0.05.

In the PCA, all participants (n = 410) from the four studies were categorized into two clusters, namely ET-B (n = 369) and Halomonas (n = 41, ET-H) (Figure 1A). The samples with ET-H were too small to evaluate for further analysis, and the analysis was conducted on participants in the ET-B group. In the present study, we analyzed the gut microbiota composition and metagenome function in the participants with MCI and AD in the ET-B group. The number of participants in the healthy, MCI, and AD groups was 125 (33.9%), 141 (38.2%), and 103 (27.9%) in ET-B and 28 (68.3%), 6 (14.6%), and 7 (17.1%) in ET-H, by χ2 test (*p* < 0.01; Figure 1A). The relative abundance of fecal bacteria in the ET-B and ET-H groups is shown in Figure 1B,C, respectively. In the ET-B participants, the relative abundance of *Bacteroidaceae, Phocaeicola, Halomonadaceae, Pseudomonadaceae, Enterobacteriaceae*, and *Streptococcaceae* decreased in the order of the healthy (n= 125), MCI (n = 141), and AD (n = 103) groups (Figure 1B; *p* < 0.001). However, there was a higher relative abundance of *Lachnospiraceae, Oscillospiraceae*, and *Bifidobacteriaceae* in the MCI group than in the AD group in the ET-B participants (Figure 1B; *p* < 0.001). In the participants in the ET-H group, the relative abundance of *Halomonadaceae, Pseudomonadaceae, Moraxellaceae*, and *Microbacteriaceae* was markedly lower, and that of *Enterobacteriaceae, Rhizobiaceae*, and *Bacillaceae* was higher in the healthy group (n = 28) than in the MCI (n = 6) and AD groups (n = 7; Figure 1C; *p* < 0.001). The relative abundance of *Enterobacteriaceae* and *Rhizobiaceae* was higher in the MCI group than in the AD group in the ET-H participants (Figure 1C; *p* < 0.001).

### 2.2. α-Diversity and β-Diversity in the Participants with ET-B

**Figure 2:**
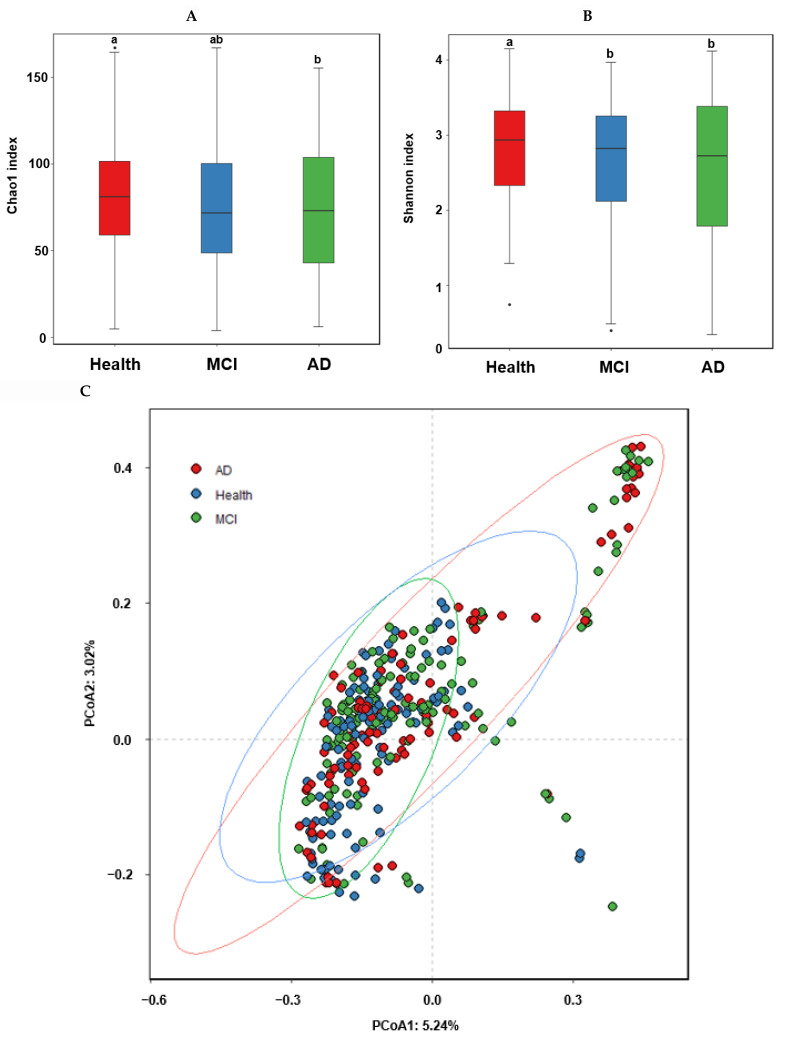

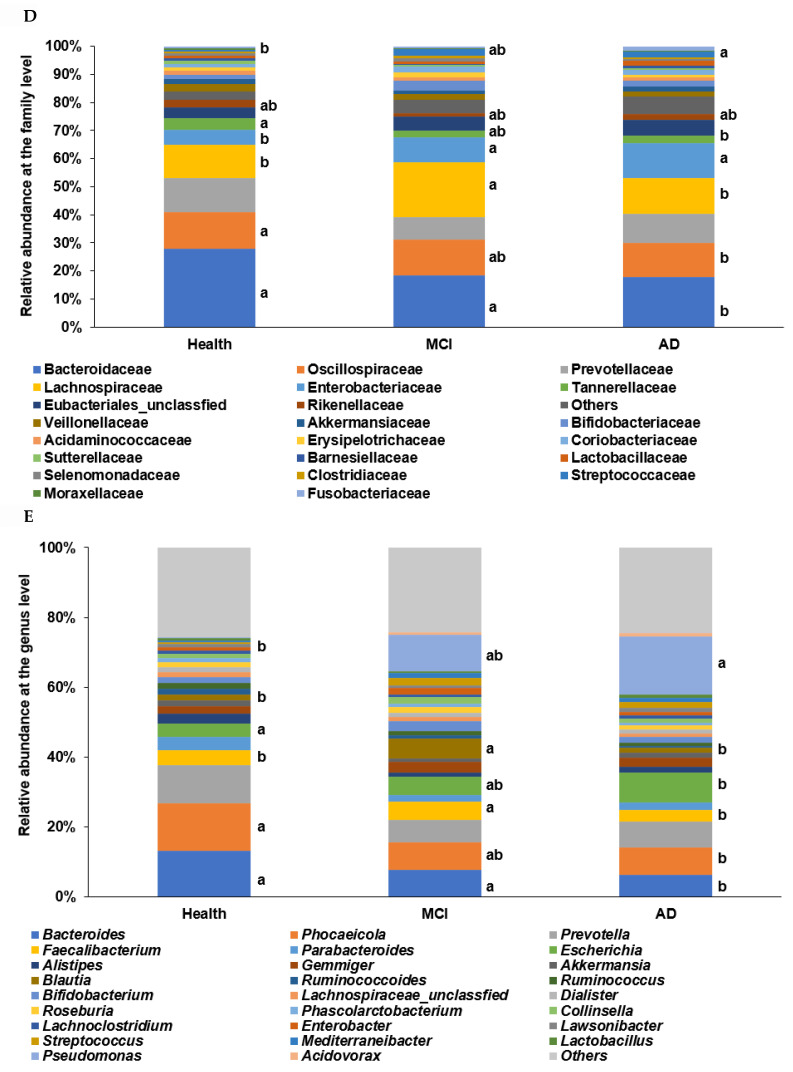
α-diversity, β-diversity, the relative abundance of the fecal bacteria according to the healthy, MCI, and AD groups in the participants with enterotype Bacteroides. (**A**) Chao1 index. (**B**) Shannon index. (**C**) B-diversity. Color of the circles represented the same as group names. (**D**) Relative abundance of the bacteria at the family level. (**E**) Relative abundance of the bacteria at the genus level. ^a,b^ Different letters on the bar indicated significant differences among the groups at *p* < 0.05.

The α-diversity represents species richness, dominance, and evenness by different indices. The observed OTUs represent the number of species per sample, while the Chao1 and Shannon indices estimate the richness of the species present in the sample. The Chao1 index was higher in the healthy group than in the MCI and AD groups, while the Shannon index was higher in the ascending order of AD, MCI, and Heath groups of the ET-B participants (Figure 2A,B; *p* < 0.05).

In the β-diversity analysis, OTUs varied among the healthy, MCI, and AD groups of the ET-B participants in the PCoA analysis (Figure 2C), and they were statistically significantly different among the groups in the Analysis of Molecular Variance (AMOVA) analysis (*p* < 0.001). At the family level of the ET-B participants, Bacteroidaceae, Halomonadaceae, and Prevotellaceae were lower. However, Enterobacteriaceae, Pseudomonadaceae, and Streptococcaceae increased in the order of the healthy, MCI, and AD groups (*p* < 0.001; Figure 2D). Lachnospiraceae increased only in MCI compared to the other groups. At the genus level, *Bacteroides, Phocaeicola, Prevotella, Ruminococcus*, and *Parabacteroides* were lower, and *Escherichia, Enterobacter, and Pseudomonas* were higher in the order of the healthy, MCI, and AD groups (*p* < 0.001; Figure 2E). Interestingly, *Faecalibacterium* and *Blautia* were higher in the MCI group compared to the others.

### 2.3. Primary Fecal Bacteria in Each Group by XGboost and SHapley Additive exPlanations (SHAP) Analysis in the Participants with ET-B

**Figure 3:**
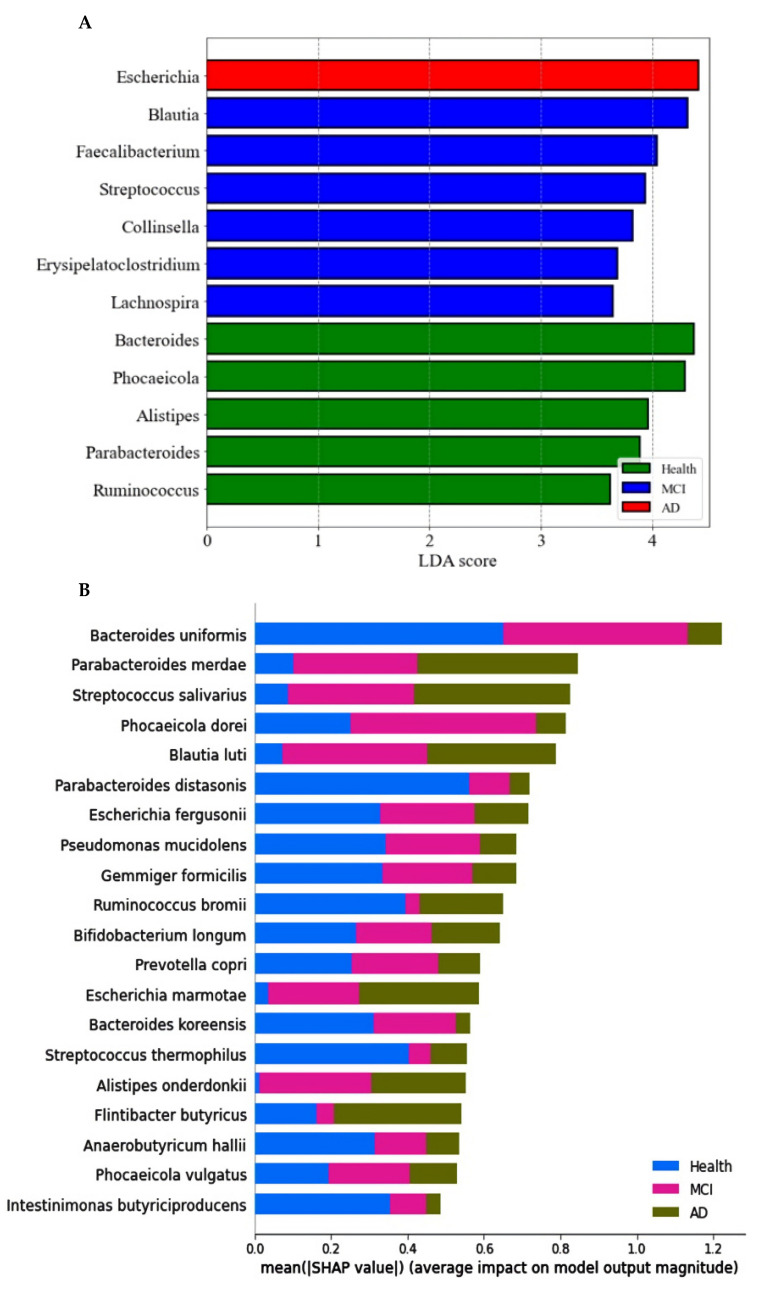

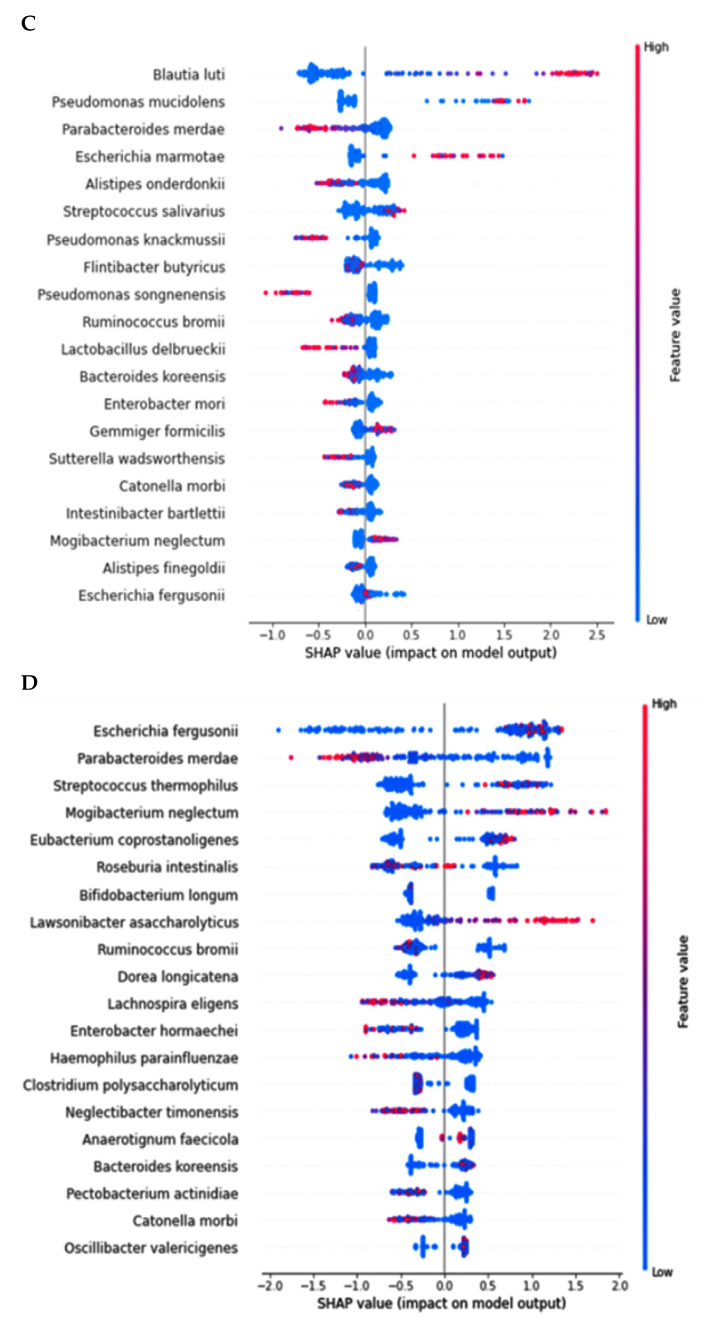
Primary bacteria selected at the genus level by the LDA and the species level by XGboost, according to the healthy, MCI, and AD groups in the participants with enterotype Bacteroides. (**A**) Linear discriminant analysis (LDA) scores of the bacteria at the genus level. (**B**) Relative abundance of bacteria at the species level, according to the healthy, MCI, and AD groups. (**C**) The predicting model for bacteria at the species level in the comparison between the healthy and MCI groups (AUC of ROC = 0.935, 10-fold crossover for trained and test sets = 0.78 ± 0.02 and 0.85 ± 0.03, respectively). (**D**) The predicting model for bacteria at the species level in the comparison between the healthy and AD groups (AUC of ROC = 0.947, 10-fold crossover for trained and test sets = 0.77 ± 0.03 and 0.81 ± 0.03, respectively).

The primary bacteria were found at the genus level with a linear discriminant analysis (LDA) score. The LDA scores of bacteria higher than three were as follows: *Bacteroides, Phocaeicolas, Alistipes, Parabacteroides*, and *Ruminococcus* in the healthy group, *Blautia, Faecalibacterium, Streptococcus, Collinsella, Erysipelatoclostrodium*, and *Lachnospira* in the MCI group, and *Escherichia* in the AD group (Figure 3A).

The prediction model for the healthy, MCI, and AD groups was generated from the relative importance of species in the fecal bacteria by Xgboost in ET-B. As seen in Figure 3B, the relative abundance of 20 species was different among the healthy, MCI, and AD groups. It showed the bacteria with different relative abundance among three groups: *Bacteroides uniformis, Parabacteroides merdae, Streptococcus salivarius, Blautia luti, Escherichia fergusonii,* and others (Figure 3B). The different relative abundance between the three groups was difficult to compare among the groups.

The relative abundance of bacteria in the healthy group was compared with that of the MCI or AD groups. In the comparison between the healthy and MCI groups, the area under the curve (AUC) of the ROC was 93.5%, and the 10-fold accuracy of the trained and test sets was 0.78 ± 0.02 and 0.85 ± 0.03, respectively (Figure 3C). The participants in the MCI group had a higher relative abundance of *Blautia luti, Streptococcus salivarius, Desulfovibrio simplex, Escherichia marmotae, Bacteroides faecis,* and *Gemmiger formicilis* than the healthy group in the healthy-MCI model (Figure 3C). In the model for the healthy-AD group comparison, the AUC of the ROC was 94.7%, and the 10-fold accuracy of the trained and test sets was 0.77 ± 0.03 and 0.81 ± 0.03, respectively (Figure 3D). *Escherichia fergusonii*, *Streptococcus thermophilus*, *Mogibacterium neglectum*, *Kawsonibacter asaccharolyticus*, and *Dorea longicatena* were higher in the AD group than in the healthy group (Figure 3D). At the same time, *Parabacteroides merdae*, *Lachnospira eligens*, *Enterobacter hormaechei*, and *Catinella morbi* were lower in the AD group than in the healthy group (Figure 3D).

### 2.4. Network of Fecal Microbiota and Metagenome Function in the Participants with ET-B

**Figure 4:**
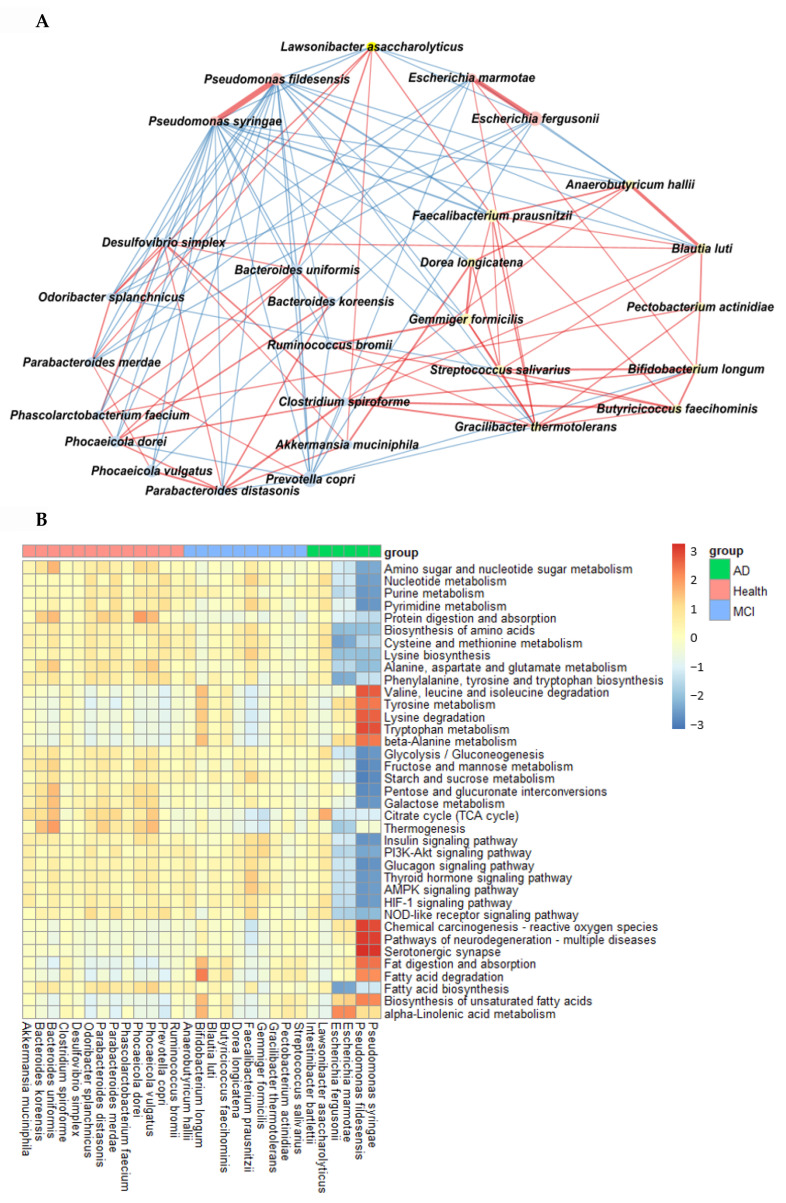
Network analysis and metagenome functions of the primary bacteria among the healthy, MCI, and AD groups (**A**). The network of the primary bacteria among the primary bacteria, according to the healthy, MCI, and AD groups in the participants with enterotype Bacteroides. Red and blue lines indicated positive and negative correlations with the Pearson correlation coefficient >0.1, respectively. (**B**) Metagenome functions of the primary bacteria in human samples.

In AD, *Pseudomonas fidesensis*, *Pseudomonas syringae*, *Escherichia marmotae*, and *Escherichia fergusonii* showed a high positive correlation in the AD group (*p* < 0.001; Figure 4A). However, they were negatively correlated with the bacteria in the MCI and healthy groups. The negative correlation was higher in the healthy group than in the MCI group. However, *Lawsonibacter asaccharolyticus* had a negative correlation with four bacteria in the AD group and a positive correlation with the MCI and healthy groups, although they belonged to the AD networking (Figure 4A). The bacteria in the healthy group were positively correlated with each other. Most bacteria in the healthy group were negatively correlated with the primary bacteria, such as *Pseudomonas fidesensis*, *Pseudomonas syringae*, *Escherichia marmotae*, and *Escherichia fergusonii* in the AD group. Some bacteria in the healthy group were also negatively correlated with those in the MCI group, including *Gracilibacter thermotolerans*, *Bifidobacterium longum,* and *Streptococcus salivarius* (Figure 4A). These results suggested that the gut bacterial network in the healthy group might protect against the survival of AD-related gut bacteria. However, the protection by the bacterial networking in MCI was too weak to move toward an AD state quickly. Therefore, a robust bacteria network can prevent the increase in harmful bacteria that could result in AD. The primary bacteria in the healthy participants can protect against AD induction by improving the gut microbiota-brain axis.

The metabolism of nucleotide, purine, and pyrimidine was negatively correlated with the primary bacteria in the AD group but positively correlated with those in the healthy group (Figure 4B). the protein digestion and most amino acid metabolisms, such as cysteine, lysine, alanine, aspartate, and glutamate metabolism exhibited a significant negative correlation with the bacteria in the AD group, while they had a positive correlation with those in the healthy group (Figure 4B). However, the metabolism of valine, leucine, isoleucine, tyrosine, tryptophan, and β-alanine was positively correlated with the bacteria in the AD group, which was in contrast to that seen with the other amino acids. The metabolism of starch, sucrose and glucose, fat biosynthesis, and glucose-related pathways, such as the insulin and glucagon signaling pathways, were significantly negatively correlated with the main bacteria in the AD group and positively correlated with those in the healthy group (Figure 4B). However, fat degradation, digestion, and absorption were significantly positively correlated with the primary bacteria in the AD group, but negatively correlated with those in the healthy group (Figure 4B).

### 2.5. Memory Deficit in the Animal Study

**Figure 5:**
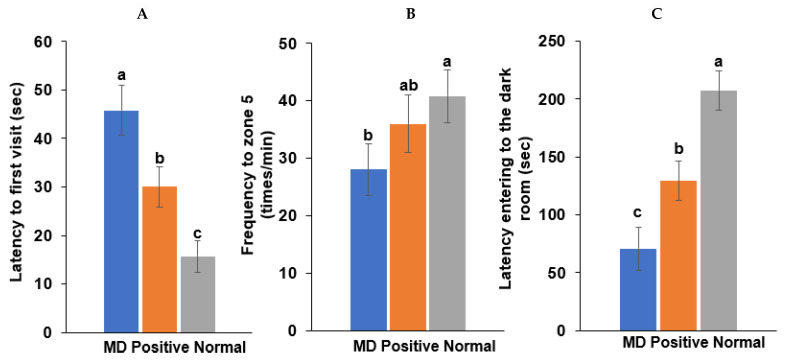
Memory function by the water maze and passive avoidance test in scopolamine-injected rats. (**A**) Latency time to first visit zone 5 in the water maze. (**B**) Frequency to zone 5 in the water maze. (**C**) Latency time entering the dark room in the passive avoidance test. Bars represent the means and standard deviations (n = 10). ^a,b,c^ Different letters on the bar indicated significant differences among the groups at *p* < 0.05 in each figure.

A spatial memory deficit determined by the water maze test was induced in the MD group (intraperitoneal scopolamine injection; n = 10), compared to the normal group (intraperitoneal saline injection; n = 10). The latency time of the first visit to zone 5, where the platform was located, was longer in the MD group than in the positive (intraperitoneal scopolamine injection plus donepezil intake; n = 10) and normal groups in the water maze test (*p* < 0.001; Figure 5). The frequencies visiting zone 5 were also fewer in the MD than in the normal (*p* < 0.05; Figure 5) group.

Following the first two training sessions in the passive avoidance tests, the short-term memory was measured in the third session. The latency time to enter the dark room was reduced in the order of the normal, positive, and MD groups in the third session (*p* < 0.001; Figure 5). The results suggested that the MD rats exhibited short-term and spatial memory impairment, compared to those in the positive and normal groups. However, the memory improvement in the positive group was lower than in the normal group.

### 2.6. Fecal Bacterial Analysis in the Animal Study

**Figure 6:**
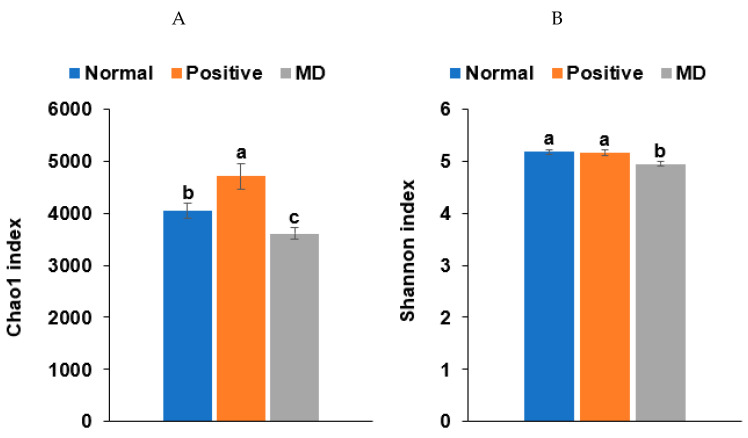

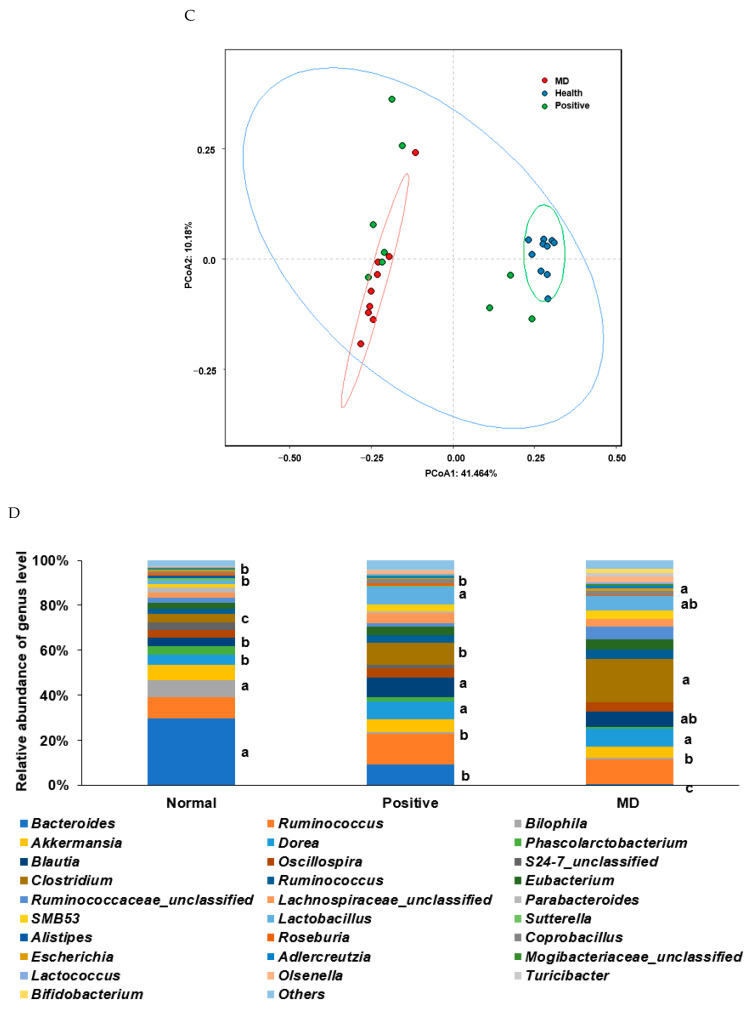

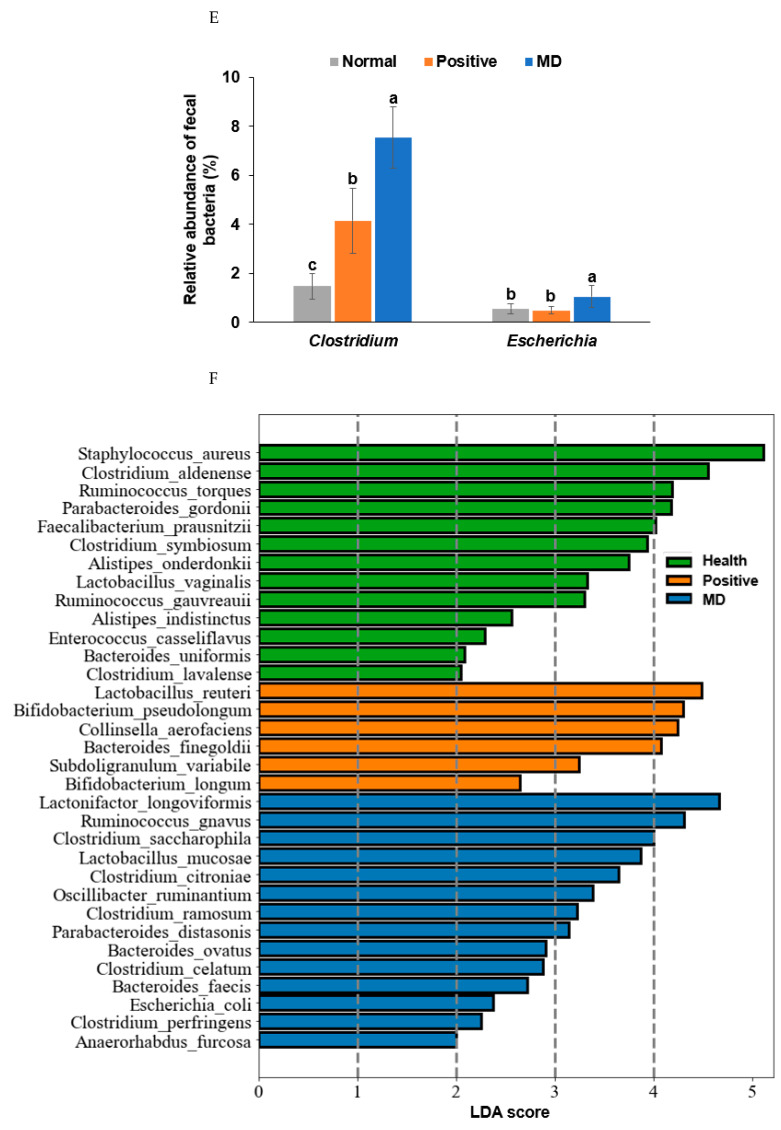

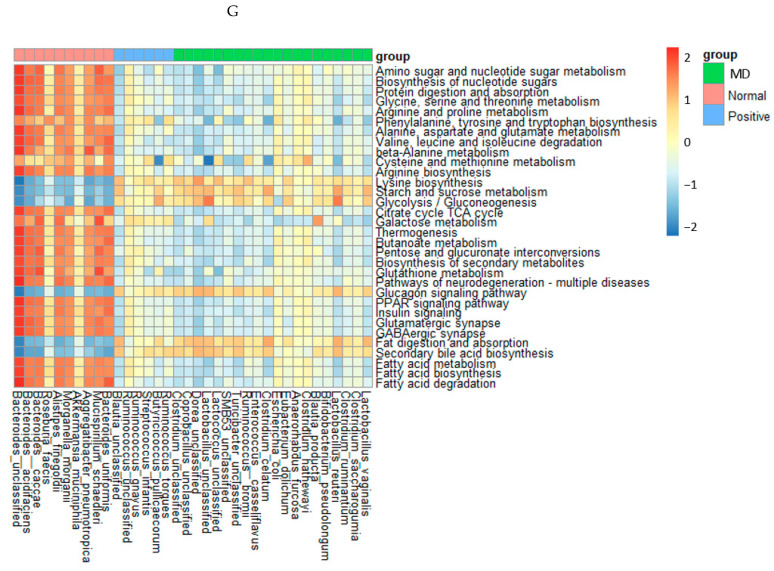
Characteristics of the fecal bacteria in the scopolamine-injected rats. (**A**) Chao1 index. (**B**) Shannon index. (**C**) β-diversity. Color of the circles represented the same as group names. (**D**) Relative abundance of the bacteria at the genus level. (**E**) LDA scores of bacteria at the species level. (**F**) Metagenome functions of the primary bacteria. (**G**) Metagenome functions of the primary bacteria in animal samples. ^a,b,c^ Different letters on the bar indicated significant differences among the groups at *p* < 0.05 in each figure.

The Chao1 and Shannon indices of the fecal bacteria, representing the α-diversity, were lower in the MD than in the normal group (*p* < 0.001; Figure 6A,B). The positive group (donepezil intake) increased the Chao1 index, compared to the MD group, suggesting the prevention of a decreased α-diversity of the gut bacteria by scopolamine injections (Figure 6A,B). The fecal bacteria were clearly clustered into three groups in the PCoA, indicating that the bacterial species were different in the three groups (*p* < 0.01; Figure 6C).

Rats fed on a high-fat diet exhibited a gut microbiota composition similar to ET-B. At the genus level, the relative abundance of *Bacteroides* and *Biophilia* was much higher, and that of *Escherichia, Blautia*, and *Clostridium* was much lower in the normal group than in the MD group (Figure 6D). The changes in these bacteria were shown in the positive group, but some protection was detected. *Lactobacillus* and *Blautia* increased in the positive, compared to the normal group (Figure 6D). The relative abundance of *Clostridium* was much higher in the MD group than in the normal group, and it decreased in the positive group than in the MD group (*p* < 0.001; Figure 6E). The relative abundance of *Escherichia* was also higher in the MD group than in the normal group and it decreased in the positive as much as the normal group (*p* < 0.05; Figure 6E). The primary bacteria in each group were used to calculate the LDA scores of the bacterial species, the LDA scores of *Staphylococcus aureus*, *Clostridium aldenense*, *Ruminoccocus torques*, *Faecalibacterium pranunsnizii*, *Clostridium symbiosm*, *Lactobacillus vaginalis*, *Ruminococcus fauvreauii*, *Bacteroides uniformis* were higher in the normal group than the other groups (Figure 6F). The relative abundance of *Ruminoccocus gnavus*, *Clostridium saccharophila*, *Lactobacillus mucosae*, *Clostridium citroniae*, *Clostridium ramosum*, *Parabacteroides distasonis*, *Bacteroides ovatus*, *Clostridium celatim*, *Escherichia coli*, and *Clostridium perfringens* was higher in MD (Figure 6F). Therefore, different species in the same genus were either beneficial or harmful for the development of the disease, and although *Clostridium* and *Escherichia* were mainly harmful to MD, some species may inhibit MD from developing by interacting with other bacteria.

### 2.7. Metagenome Function of the Fecal Bacteria in the Animal Study

The metagenome results of the animals were similar to those in humans, but their correlations were stronger in animals than in humans (Figure 6G). The metabolism of amino acids, including glycine, arginine, cysteine, methionine, proline, serine, and threonine, the biosynthesis of tryptophan, tyrosine, and phenylalanine, and the degradation of valine, isoleucine, and leucine were much lower in the MD group than in the normal group. The metagenome pathways in the positive group were altered from those in the MD group and also different from those in the normal group (Figure 6G). Starch and sucrose metabolism, glycolysis, fat digestion and absorption, and secondary bile acid biosynthesis were much lower in the normal group than in the MD and positive groups (Figure 6G). Interestingly, the pathways related to AD, such as glutamatergic synapse, GABAergic synapse, peroxisome proliferator-activated receptor (PPAR-γ), and insulin signaling pathways, were reduced in the MD and positive groups, compared to the normal group. However, glucagon signaling showed the opposite pattern (Figure 6G).

## 3. Discussion

The autonomic nervous system regulates involuntary physiologic processes, such as the heart rate, blood pressure, digestion, temperature, fluid balance, and urination through the opposing actions of the sympathetic nervous system (SNS) and the PNS. The SNS is responsible for the fight-or-flight response through adrenergic synaptic neurons, while the PNS helps reduce stress and the heart rate through cholinergic synaptic neurons [17]. The autonomic dysfunction is prevalent in people with dementia, and with impairment in the PNS, especially to the cholinergic neurotransmitter system, contributes to cognitive dysfunction, leading to AD [18]. The autonomic nervous system is bidirectionally involved in the microbiota-gut-brain axis, mainly through the vagus nerve, the principal component of the PNS. It plays a role in the cross-talk between the brain and gut microbiota and delivers messages to the brain regarding the gut microbiota and related metabolites. Moreover, factors such as stress, inhibit the vagus nerve to potentiate gut inflammation and decrease intestinal permeability, possibly contributing to the modulation of the gut microbiota [11]. Therefore, suppressing the cholinergic nerve system can contribute to memory dysfunction and inflammatory bowel disease by modulating the microbiota-gut-brain axis.

The present study showed that the gut microbiota composition was different in healthy, MCI, and AD patients of the Bacteroides enterotype: *Bacteroides* and *Phocaeicola* decreased, and *Escherichia* and *Pseudomonas* increased in the MCI and AD patients. At the same time, *Bluatia* was elevated only in MCI patients. When rats were fed a high-fat diet, they elevated *Bacteroides* to mimic the Bacteroides enterotype in humans. The suppression of the PNS by scopolamine in MD group decreased *Bacteroides* and increased *Clostridium, Lactobacillus*, and *Escherichia,* compared to the normal group in the present animal study. Previous studies have demonstrated that a high-fat diet increases Bacteroidaceae, compared to a low-fat diet in rats with intact gallbladders, but does not do so in rats without gallbladders [19]. It suggests that bile acid is responsible for the increased Bacteroidaceae. The PNS inhibition suppresses the bile acid synthesis and secretion, which may change the gut microbiota in a high-fat diet [19,20]. In the present study, the reduced Bacteroidaceae in the scopolamine-injected rats, was related to inhibiting the bile acid secretion by the suppressed PNS. Therefore, the PNS inhibition develops memory impairment, linked to the gut microbiota changes.

Cognitive function is modulated through the gut-brain axis involved in the PNS (vagus nerve) activity, circulating hormones, and proinflammatory cytokines. Increased sympathetic activity suppresses the PNS, which contributes to the gut dysbiosis, elevating inflammation and sympathetic activity [21]. The PNS suppression is linked to memory impairment, which progresses to neurodegenerative diseases, including Alzheimer’s disease [18,22]. *Bacteroides* decreased, and *Clostridium, Escherichia*, and *Lactobacillus* increased in rats injected with scopolamine. Donepezil treatment (positive group) prevented the decrease of *Bacteroides* and increased *Clostridium,* but it elevated *Blautia* and *Lactobacillus* more than in the MD and normal groups. The α-diversity was lower in the MD group than in the normal group. Previous studies have shown that scopolamine injections decreased the α-diversity, increased Firmicutes, and decreased *Bacteroides* [23]. Scopolamine injections have been demonstrated to positively correlate with *Clostridium, Bifidobacterium,* Ruminococcaceae_unclassified, Lachnospiraceae_unclassified, and *Lactobacillus* while being negatively related to *Desulfovibrio, Akkermansia*, and *Blautia* [23]. Scopolamine injections increase the intestinal permeability and induce memory impairment [23,24]. The present study also showed similar changes in the gut microbiota and memory function. Therefore, the changes in the fecal bacterial composition were associated with suppressing the PNS to increase the intestinal permeability and decrease the digestive fluids, especially bile acids. Moreover, donepezil partially prevents the PNS inhibition by scopolamine injections.

The primary gut bacteria in the different groups can be determined by LDA, which identifies the bacteria that are more abundant in one group than another, adopting a principle of analysis closely related to the principal component analysis and linear regression analysis [24]. The present study demonstrated that only *Escherichia* was selected with a high LDA score in the AD group. Moreover, in the SHAP analysis using XGboost, *Escherichia* and *Pseudomonas* were high in the AD group and belonged to Proteobacteria and Gammaproteobacteria, respectively. The bacteria infect the host through the consumption of infected foods. They cannot grow in healthy people, due to their healthier gut conditions. However, they can grow in certain conditions, such as the reduced secretion of digestive juices, especially bile acid, and an increased intestinal permeability related to a suppressed PNS [25]. In AD patients, the serum concentration of bile acid metabolites is modulated, compared to elderly persons without AD [25,26,27]. It suggests that AD bidirectionally increases harmful bacteria, namely *Escherichia* and *Pseudomonas*, due to the disturbed microbiota-gut-brain axis. However, the primary bacteria in the MCI group were included from both AD and healthy groups, suggesting MCI can be progressed into AD and be prevented by modulating the gut microbiota.

Some beneficial gut bacteria in the MCI group could prevent or delay the progression to AD, but other harmful bacteria could lead to the progression to AD. Previous studies have reported that having an *Escherichia coli* infection increases the risk of AD by 20.8 (95% CI = 17.7–24.3) times [27], and the genus *Escherichia* increases in both fecal and blood samples of AD and MCI patients [28]. *Pseudomonas aeruginosa* is linked to the AD pathogenesis and is believed to promote amyloid-β deposition, called amyloidosis [29,30]. Indeed, the amyloid-associated pathogenesis of AD may be triggered by a shift in the gut microbiota from MCI to AD. *Escherichia coli* K99 pili protein and lipopolysaccharide (LPS) have been shown to be co-localized in the amyloid plaque in the postmortem brain tissues of AD patients [28,31]. The present study also demonstrated that a relative abundance of *Escherichia* was observed in the descending order of the AD, MCI, and healthy groups. A lower Escherichia in the MCI and healthy groups could be due to beneficial bacteria, such as *Faecalibacterium, Butyricicoccus faecihominis,* and *Bifidobacterium longum*, which produce butyrate, preventing its increase.

The network analysis represents the predominant bacteria with a co-occurrence and positive and negative relations among the groups [32]. The bacteria with positive connections grow and survive with each other, the harmful bacteria are unable to cause infections, and the bacteria with negative connections find it difficult to live together [33]. Therefore, the harmful bacteria can be eliminated by increasing the beneficial bacteria’s negative connection with harmful bacteria [33]. In the present study, the network analysis showed that the *Escherichia marmotae, Escherichia fergusonii, Pseudomonas fidesensis*, and *Pseudomonas syringae* were the primary gut bacteria in the AD group, and they were negatively associated with the predominant gut bacteria in the healthy and MCI groups. However, the number of bacteria with negative connections in the AD group was much higher in the co-occurrent bacteria of the healthy group than in the MCI group. The present network analysis results can be applied to explore a therapeutic approach for preventing or delaying the progression of MCI and AD by modulating the gut microbiota.

The present study was novel in its intention to show potential therapeutic approaches to prevent and delay the AD progression by modulating the gut microbiota with a higher power. Most studies on the gut microbiota are based on small sample sizes, and their power can be weak. The present study included several studies and combined the data to increase the power of the study. The limitations of the current study were as follows: (1) Age and gender of the participants were the only data provided with the fecal bacteria files. Lifestyles, including nutrients, smoking, alcohol consumption, medications, and antibiotic intake, play critical roles in modulating the gut microbiota [15]. However, the environmental factors could not be controlled to identify the potential confounders, since the availability of this data was limited. (2) The data were collected in case-control studies, and the results could not be applied to evaluate cause and effect. However, the observation studies supported that the microbial community changes can influence the host metabolism through their metabolite production [34]. Further studies to represent the causality between the gut bacteria and AD should be conducted. (3) In the present animal study, fecal bacteria modulation by scopolamine injections was conducted in a high-fat diet to mimic ET-B [16]. However, it needed to be studied in a normal-fat diet (25–30 energy% diet) since a high-fat diet itself can change fecal bacteria, related to memory impairment [35].

In conclusion, the fecal bacteria in the participants with the Bacteroides enterotype in the AD and MCI groups, significantly differed from those in the healthy group. The relative abundance of *Escherichia fergusonii*, *Mycobacterium neglectum,* and *Lawsonibacter asaccharolyticus* was much higher in the AD group than in the normal group. That of *Blautia, Facalibacterium, Streptococcus, Collinsella*, and *Erysipelatoclostridium* was observed to be higher in the MCI group than other groups, using the optimal model generated from the XGboost analysis. In the animal study, the relative abundance of *Bacteroides* and *Bilophilia* was lower, and that of *Blautia*, *Escherichia,* and *Clostridium* was higher in the MD group than in the healthy group. The results suggested that MCI and MD were associated with the PNS suppression, which was thought to progress into AD, eventually. MCI increased *Clostridium* and *Blautia,* and its progression to AD elevated *Escherichia* and *Pseudomonas*. Therefore, the modulation of the PNS altered the gut microbiota and brain function, potentially through the gut-brain axis. Further studies are needed to identify the therapeutic agents to enhance the activation of the PNS and decrease the harmful bacteria associated with AD, by increasing the beneficial bacteria with their inverse relationship with the harmful bacteria.

## 4. Methods

### 4.1. Data Collection for the Human Study

**Figure 7:**
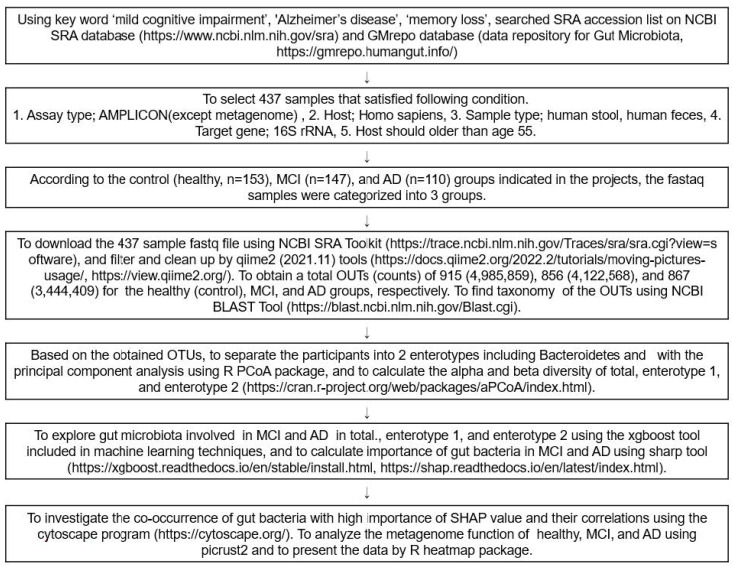
Scheme of the selection and analysis procedures of the fecal bacteria.

Figure 7 presents the overall data selection process and fecal bacteria analysis as a flow chart. FASTA and FASTAQ files of the fecal bacteria in the healthy, MCI, and AD participants were obtained through a search of the National Center for Biotechnology Information (NCBI) database (https://www.ncbi.nlm.nih.gov/; accessed on 3 December 2021), European Nucleotide Archive (ENA) browser (www.ebi.ac.uk/ena/browser/home; accessed on 10 December 2021), and data repository for Gut Microbiota (GMrepo database; https://gmrepo.humangut.info/; accessed on 21 December 2021) up to April 2022. The keywords used for the search were ‘MCI’, ‘AD’, ‘dementia’, ‘memory loss’, and ‘cognitive impairment’ in the Medical Subject Headings (MeSH) terms and a combination of the terms. The FASTA and FASTAQ from the human fecal bacteria studies on MCI and AD were selected. The inclusion criteria for the FASTA and FASTAQ files were as follows: (1) assay type, AMPLICON sequencing (Miseq); (2) host, homo sapiens; (3) sample type, human stools or human feces, (4) target gene; 16S rRNA, (5) hosts were 60–85 years old. Patients or their family members volunteered to participate in the studies, received the fecal samples, and signed the informed consent. Based on the above, four projects were found, and the corresponding Institutional Review Board approvals were taken for each. An experienced neurologist diagnosed the MCI and AD patients, using the clinical assessment protocol, stipulated by the National Institute on Aging /Alzheimer’s Association’s (NIA-AA) Diagnostic guidelines for Alzheimer’s Disease.

### 4.2. Animal Care

Thirty-eight-week-old male Sprague Dawley rats (n = 10 per group; total n = 30) were purchased from Daehan Bio Inc. (Eum-Sung, Korea). They were acclimatized for one week in an animal facility at Hoseo University. Each animal was housed in an individual stainless-steel cage in a controlled environment (23 °C, 12 h light/dark cycle) and was given a high-fat diet (43 energy percent diet) and water ad libitum. All procedures conformed with the Guide for the Care and Use of Laboratory Animals (8th edition) issued by the National Institutes of Health (Washington DC, USA) and were approved by the Institutional Animal Care and Use Committee of Hoseo University (HSIACUC-201836).

### 4.3. Experimental Design for the Animal Study

**Figure 8:**
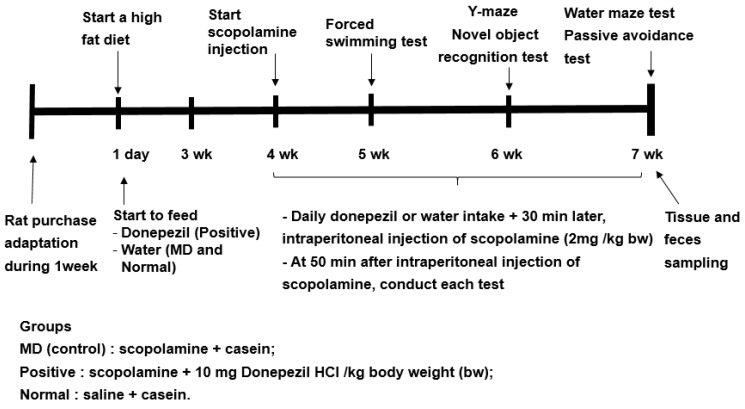
Experimental design for the animal study.

Previous studies have shown to increase in the relative abundance of Bacteroides in animals and humans fed a high-fat diet [16]. Since the fecal bacteria of the participants in the present study mainly belonged to ET-B, all rats were fed a high-fat diet to mimic the fecal bacteria, such as the ET-B. The experimental design is presented in Figure 8. The rats were divided into the memory deficit (MD), positive, and normal groups. Scopolamine and donepezil (Sigma Aldrich, St. Louise, MO, USA) were dissolved in 0.9% saline and water, respectively. The rats in the positive group were orally administered donepezil at 1 mg/kg body weight/day using a feeding needle, and those in the MD and normal groups were given water for seven weeks. The dosage of donepezil (10 mg/kg bw/day) was assigned, based on the previous studies [36]. Scopolamine dosage (2 mg/kg bw/day) and period (50 min later measurement) to induce the memory impairment were designated from our preliminary study. At the beginning of the fourth week, a rat daily had an oral administration of water or donepezil in the MD and positive groups, respectively, and then 30 min later, had an intraperitoneal injection of scopolamine (2 mg/kg body weight/day). The rats in the normal group were intraperitoneally injected with saline without the induction of memory deficit. At the end of the experiment, the fecal samples were collected from the cecum of all rats.

### 4.4. Memory Assessment Using the Passive Avoidance Test and the Morris Water Maze Test in the Animal Study

The passive avoidance apparatus is equipped with a two-compartment dark/light shuttle box, wherein the rat quickly enters the darkroom when left in the light shuttle box [37]. Electrostimulation (75 V, 0.2 mA, 50 Hz) is delivered to the feet of the rat when entering the dark room to train the rat not to enter the dark room. The rat is trained in this manner in two acquisition trials lasting 8 h. Then, after 16 h, i.e., after the second trial, the time latency to enter the dark chamber is reassessed in the same manner but without electrical stimulation. Latency is checked up to a maximum of 600 s. The longer the latency time, the better the memory function.

The spatial memory function is evaluated using the Morris water maze test, which assesses the hippocampal-dependent learning, including the acquisition of spatial memory [38]. At the start, a rat was placed at zone 1 of the pool and then began to find the platform at zone 5 [37,39]. The water maze test was conducted three times: the rat learned to find the platform located in zone 5 on days 1 and 2, and 5, and the first two sessions were the training sessions. The latency time, frequency to visit, and duration in zone 5 at the third session were measured to evaluate spatial memory. The test was performed with a cut-off time of 600 s.

### 4.5. Fecal Bacteria Sequencing for the Animal Study

The investigation of the fecal microbiome communities was carried out using cecum feces by Miseq using next-generation sequencing (NGS) technology [40]. Bacterial DNA was extracted from the feces of the rats, and the sequencing was performed, using the Illumina MiSeq standard operating procedure and a Genome Sequencer FLX plus (454 Life Sciences) (Macrogen, Seoul). The DNA was amplified with 16S amplicon primers in the V3-V4 region by PCR, and libraries were prepared for the PCR products, according to the GS FLX plus library prep guide, as described previously [41].

### 4.6. Fecal Bacterial Community Analysis

The fecal FASTAQ files from humans were downloaded from 410 participants using the National Center for Biotechnology Information (NCBI) Sequence Read Archive (SRA) toolkits (https://trace.ncbi.nlm.nih.gov/Traces/sra/sra.cgi?view=software; accessed on 13 January 2022). The FASTAQ from the humans and animals were separately filtered and cleaned up with qiime2 tools (https://view.qiime2.org/; accessed on 10 February 2022). From the human samples, operational taxonomic units (OTUs) for the healthy, MCI, and AD groups were obtained, respectively, while from the animal studies, the normal, positive, and MD groups, respectively. Using the Qiime2 program, 410 sequences were obtained after merging the double-ended sequences with the “make.contigs” command; the sequences were aligned with the SILVA v 1381 database, and the non-target sequences, such as mitochondria, archaea, fungi, and unknown sequences were removed. The remaining sequences were preclustered, and the chimeras were eliminated, using the “chimera.vsearch” command [36]. The sequences were then clustered with 97% similarity. The taxonomy of the OTUs in the FASTAQ files was annotated, according to the NCBI Basic Local Alignment Search Tool (BLAST) (https://blast.ncbi.nlm.nih.gov/Blast.cgi; accessed on 24 February 2022). Finally, 40,289 representative sequences were obtained for the subsequent analyses, and their biome files containing the taxonomy and counts were used for further analysis.

### 4.7. Enterotypes

The enterotypes were classified using the taxonomy and counts of the fecal bacteria in the human fecal FASTA/Q samples, including the control (healthy, n = 153), MCI (n = 147), and AD (n = 110) groups, by the principal component analysis (PCA). The number of enterotypes was assigned, based on eigenvalues >1.5 in the PCA. Two enterotypes satisfied these eigenvalues using the FactoMineR and Factoextra packages in the R software [42]. The names of the enterotypes were assigned from their primary bacteria: The main bacteria in enterotypes 1 and 2 were Bacteroidaceae and Halomonadaceae, called ET-B and ET-H, respectively. ET-B and ET-H included 369 and 41 participants, respectively. The number of participants with ET-H was too small to analyze the fecal bacteria; hence, the fecal bacteria in ET-B were used to identify MCI- and AD-related bacteria.

### 4.8. α-Diversity, β-Diversity, and LDA Scores

Alpha-diversity (α-diversity) is the species diversity in a site at a local scale, β-diversity is the ratio between the regional and local species diversity, and the LDA scores represent the effect size of each abundant species. The α-diversity metric was calculated with a “summary.single” command in the Mothur software package, and the Chao1 and Shannon indices were obtained. For the β-diversity measurement, the clearcut command in Mothur was used to construct a phylogenetic tree, the “unifrac.unweighted” command was applied to calculate the unweighted UniFrac distance matrix, and then the principal coordinate analysis (PCoA) was used for the visualization. The AMOVA command was used to compare the significant differences among the β-diversity groups. The LDA scores were analyzed with the lefSe command in the Mothur program.

### 4.9. XGBoost Classifier Training and the SHAP Interpreter

The fecal bacteria compositions of the *Bacteroides* (ET-B) participants in the healthy, MCI, and AD groups were analyzed. The characteristic features included the relative abundance of the fecal bacteria at the species level, the anthropometric variables, the serum biochemical, and metabolic variables. The fecal data were divided randomly into 80% (n = 295) for the training set and 20% (n = 74) for the testing set. A random grid search was used to find the best hyperparameter settings, and the search was carried out 1000 times in the XGBoost algorithm [43]. We first trained the XGBoost algorithm with all of the variables to find the top 10 most important variables and then used these ten variables to retrain the XGBoost algorithm. The best model with the highest receiver operating characteristic (ROC) area, accuracy, and 10-fold cross-validation in the test data set was selected from the random forest and XGBoost algorithm models. The 10-fold cross-validation was calculated using the cross_val_score function in the sklearn package. The function split the original training data into ten subsets and alternately used nine as the training data and one as test data for iterating ten times. Finally, ten sets of data were generated to obtain the mean and variance, which were used as the final accuracy result of the model. The 0.9 value of the 10-fold cross-validation indicated that the accuracy of the selected model was 90%.

The SHAP analysis is a method used to explain the output of the XGBoost model [44]. We used the SHAP (0.39.0) package to calculate the SHAP value of each variable relative to the classifier (diet types). We observed the importance of the variable and its impact on the classification.

The network analysis to determine the links among the gut bacteria at the species level was carried out using the Cytoscape program, downloaded from the website (https://cytoscape.org/; accessed on 9 March 2022). 

### 4.10. Metagenome Function of the Fecal Bacteria by Picrust2

The metabolic functions of the fecal bacteria were estimated from the genes they contained. They were determined, using the FASTA/Q files and count tables of the fecal bacteria on Phylogenetic Investigation of Communities by Reconstruction of Unobserved States (Picrust2), a software for predicting functional abundances, based only on marker gene sequences [45]. The metabolic functions were based on the Kyoto Encyclopedia of Genes and Genomes (KEGG) Orthologues (KO), mapped using the KEGG mapper (https://www.genome.jp/kegg/tool/map_pathway1.html; accessed on 30 March 2022) [40]. The gut microbiome was used to explore the differences in the metabolic functions among the groups.

### 4.11. Statistical Analysis

The statistical analysis was performed using SAS version 7 (SAS Institute; Cary, NC, USA) and the R package. The data were expressed as the mean ± standard deviation (SD), and statistical significance was set at *p* < 0.05. Multiple comparisons were conducted with Tukey’s test when the three groups had statistically significant differences. Visualization of the data was conducted using R-studio and the ggplot2 package.

## Figures and Tables

**Table 1:** Characteristics of human fecal samples used in the present study.

Study	Design	Age (Year)	Country	n	DNA Extraction	16S Region	Sequencing Method	Accession Number
Khine WWT et al.	Case-Control	60–85	Singapore	93	Quanti-iT ™ PicoGreen^®^	V3-V4	Miseq	PRJEB32675
Li Z et al.	Case-Control	63–66	China	179	DNA	V3-V4	Miseq	PRJNA489760
Liu P et al.	Case-Control	70–77	China	13	DNA extraction kit	V3-V4	Miseq	PRJNA496408
Yıldırım S et al.	Case-Control	67–71	Turkey	125	QiaAmp DNA stool minikit	v2	Miseq	PRJNA734525

## Data Availability

The authors confirm that the data supporting the findings of this study are available within the article and its Supplementary Materials. The FASTA/Q files for human studies were downloaded from the NCBI, and the data for the animal studies were available upon request to the corresponding author.

## Supplementary Materials

The following supporting information can be downloaded at: https://www.mdpi.com/article/10.3390/ijms232113574/s1.
