# Supplementary material for: Modulation of the Gut Microbiota in Memory Impairment and Alzheimer’s Disease via the Inhibition of the Parasympathetic Nervous System
Source: Int J Mol Sci. 2022 Nov 5;23(21):13574. doi: 10.3390/ijms232113574 (PMC9657043; doi:10.3390/ijms232113574)
Supplement: Supplementary file 1 [file ijms-23-13574-s001.zip › ijms-2002832-supplementary.pdf]

Supplementary Figure S1. Fecal bacteria composition of healthy participants according to enterotypes in each country

A. China

B. Singapore

C. Turkey

Enterotypes were classified using the taxonomy and counts of fecal bacteria in the fecal FASTA/Q samples of healthy participants in each country by principal component analysis (PCA). The number of enterotypes was assigned based on eigenvalues >1.5 in PCA analysis.

A.

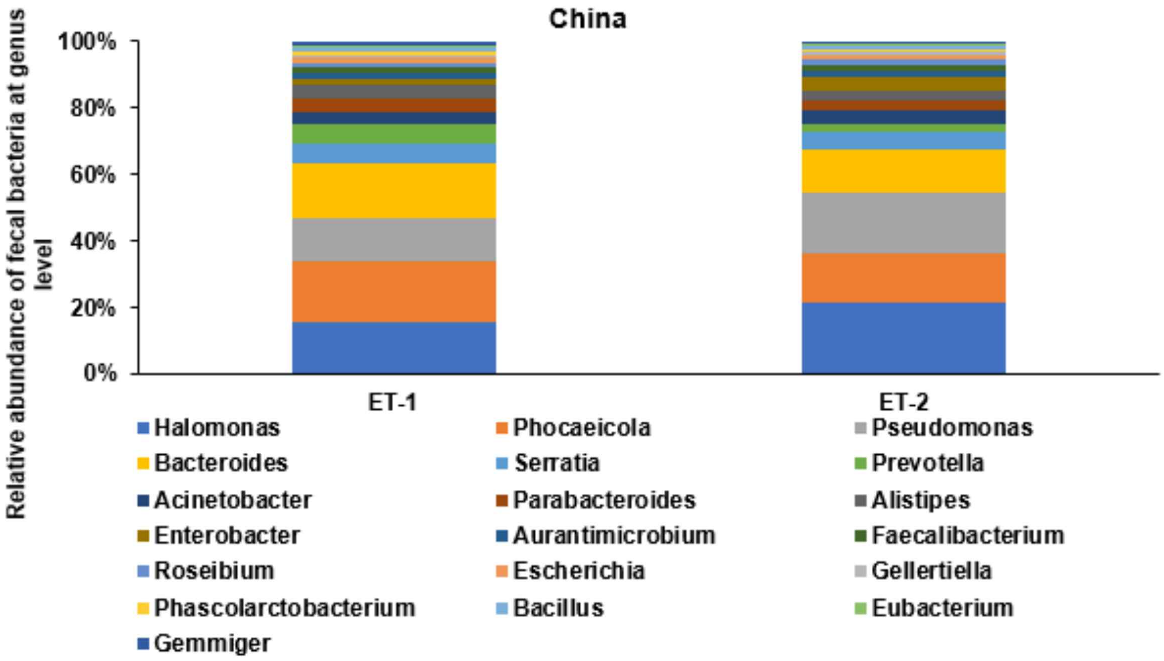

B.

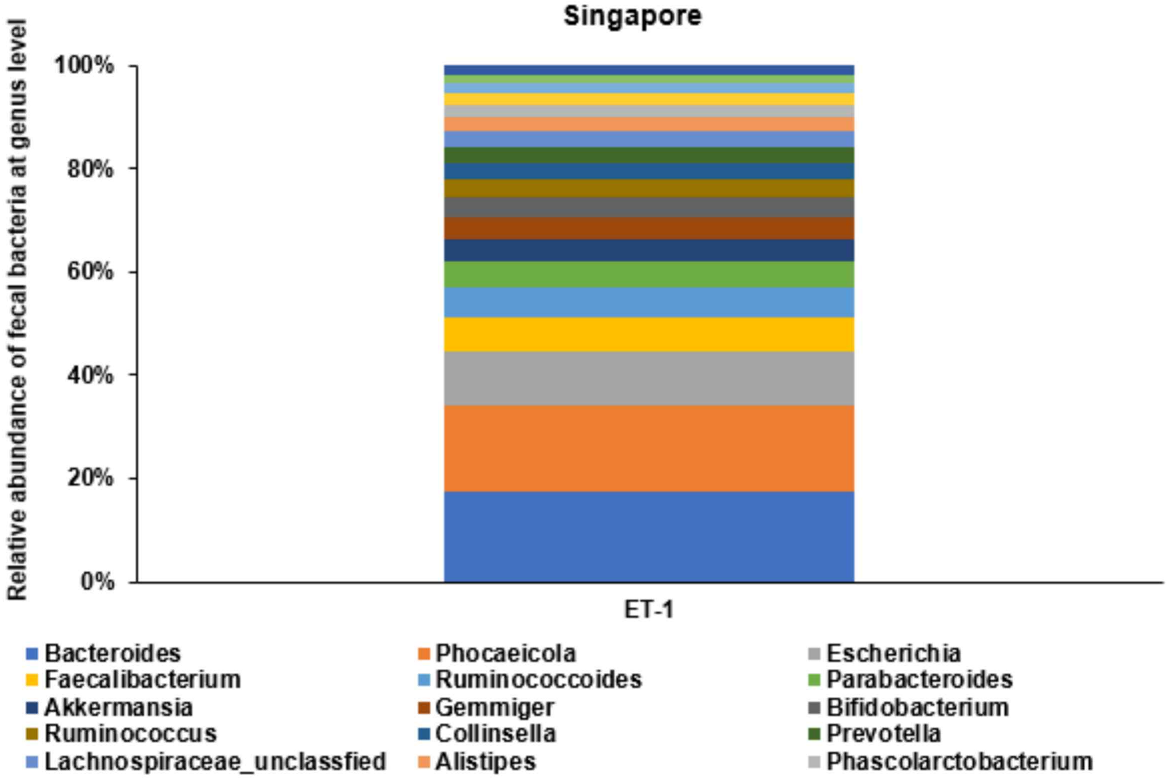
Singapore
Relative abundance of fecal bacteria at genus level
100%
80%
60%
40%
20%
0%
ET-1
Bacteroides
Phocaeicola
Escherichia
Faecalibacterium
Ruminococcoides
Parabacteroides
Akkermansia
Gemmiger
Bifidobacterium
Ruminococcus
Collinsella
Prevotella
Lachnospiraceae_unclassfied
Alistipes
Phascolarctobacterium

C.

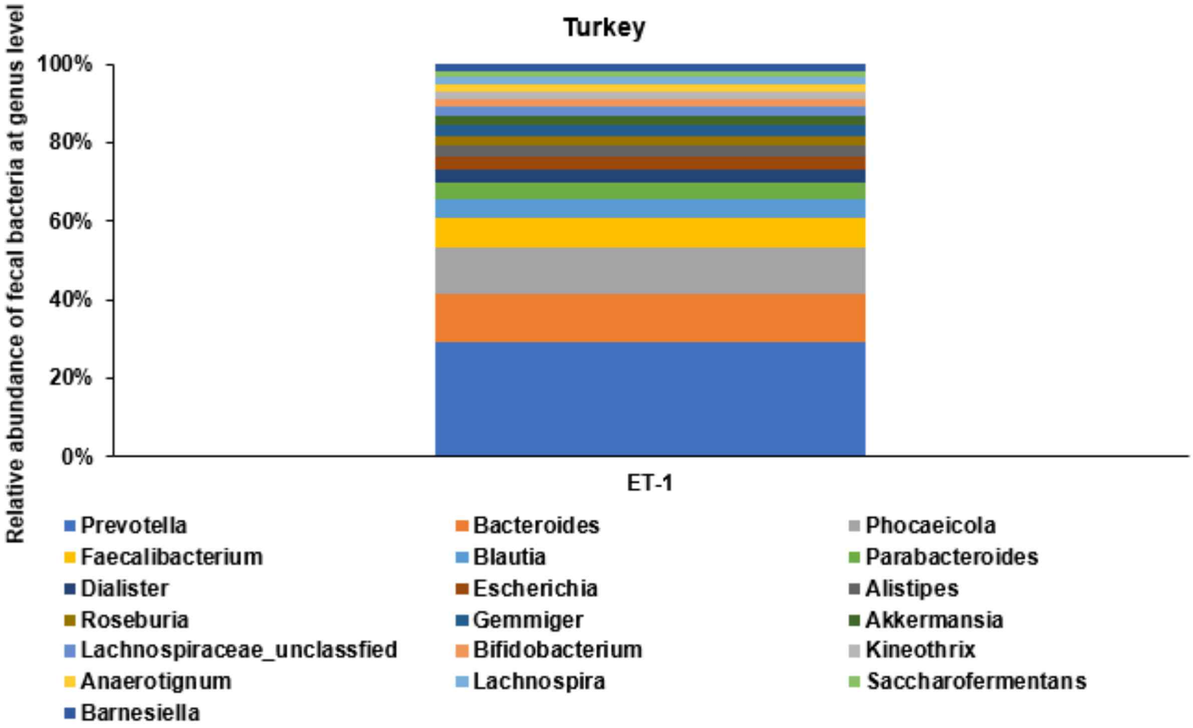
Turkey
Relative abundance of fecal bacteria at genus level
100%
80%
60%
40%
20%
0%
ET-1
Prevotella
Bacteroides
Phocaeicola
Faecalibacterium
Blautia
Parabacteroides
Dialister
Escherichia
Alistipes
Roseburia
Gemmiger
Akkermansia
Lachnospiraceae_unclassfied
Bifidobacterium
Kineothrix
Anaerotignum
Lachnospira
Saccharofermentans
Barnesiella
